# CRISPR Del/Rei: a simple, flexible, and efficient pipeline for scarless genome editing

**DOI:** 10.1038/s41598-022-16004-w

**Published:** 2022-07-13

**Authors:** Kyra L. Feuer, Marah H. Wahbeh, Christian Yovo, Eman Rabie, Anh-Thu N. Lam, Sara Abdollahi, Lindsay J. Young, Bailey Rike, Akul Umamageswaran, Dimitrios Avramopoulos

**Affiliations:** 1 McKusick-Nathans Department of Genetic Medicine, Johns Hopkins School of Medicine, Baltimore, USA; 2 Predoctoral Training Program in Human Genetics and Molecular Biology, Johns Hopkins School of Medicine, Baltimore, USA; 3 Medical Molecular Genetics Department, Human Genetics and Genome Research Division, National Research Centre, Cairo, Egypt; 4 Biotechnology Program, School of Sciences and Engineering, The American University in Cairo, Cairo, Egypt

**Keywords:** Biochemistry, Genetics, Diseases, Medical research

## Abstract

Scarless genome editing of induced pluripotent stem cells (iPSCs) is crucial for the precise modeling of genetic disease. Here we present CRISPR Del/Rei, a two-step deletion-reinsertion strategy with high editing efficiency and simple PCR-based screening that generates isogenic clones in ~ 2 months. We apply our strategy to edit iPSCs at 3 loci with only rare off target editing.

Scarless editing, defined as the introduction of a specific change into the genome without additional mutations, is crucial for modeling disease-associated genotypes. iPSCs are a preferred cell type for disease modeling^1, 2^; however the standard one-step CRISPR/Cas9 editing approach which requires introduction of a repair template to facilitate homology directed repair (HDR) has been found inefficient in iPSCs across multiple reports that provide detailed information on efficiency and lack of additional edits. Gupta et al.^3^ reported only 4 in 2500 scarlessly edited colonies in their one-step attempts. Yang et al.^1^ similarly showed ~ 1% efficiency and Schrode et al.^4^ had to perform 2 rounds of enrichment via sib selection before reaching an efficiency of 4%. In our hands, when trying 1-step editing of SNP rs4766428 (described for 2-steps below), we screened the bulk edited cells with a restriction enzyme (Cac8I) whose site should have been abolished by editing, but could detect no visible undigested PCR product, estimating < 1% efficiency. These results support that the one-step approach is not efficient for iPSCs, unless an additional protospacer or protospacer-adjacent motif (PAM) site is also changed (a “CRISPR-blocking” mutation)^5^, which however is no longer scarless editing. Several other scarless editing approaches have been described but all have limitations. Two-step editing using large cassettes that are later excised^6–8^ is promising but involves transfection of large amounts of DNA that can be toxic to the cells and can be laborious to assemble, especially if one wants to follow identical procedures for both alleles. Base editing can be problematic due to undesired editing of other nearby bases and restriction to specific modifications^9^. Finally prime editing can be promising with efficiencies for scarless editing currently reported to reach 54% in iPSCs^10, 11^. However, this method requires extensive in vitro optimization of pegRNA functional domains, and undesired edits at the target site including indels and pegRNA integration have been reported^12, 13^. To address these limitations, we used iPSCs to develop CRISPR Deletion and Reinsertion (Del/Rei) (Fig. 1), an efficient and user-friendly scarless editing strategy. The foundation of CRISPR Del/Rei is the strategic design of three single-guide RNAs (sgRNAs) used in a two-step editing protocol. In Step 1, two sgRNAs recruit Cas9 to create a ~ 45-110 bp deletion that removes a target variant and parts of the protospacers but spares at least one PAM site (Fig. 1a–b). The disruption of the protospacers prevents further sgRNA binding and Cas9 cleavage, resulting in high editing efficiency. In Step 2, the deleted sequence is reinserted (Fig. 1b–c) with the desired variant allele(s). The preserved PAM site(s) from Step 1 and the new adjacent sequence is then used as the protospacer for the third sgRNA, which we term “synthetic” (syn-sgRNA). The reinsertion is achieved by HDR using the syn-sgRNA and a single-stranded oligodeoxynucleotide (ssODN) template. Upon reinsertion the syn-sgRNA protospacer is destroyed, resulting in no further editing and increasing HDR efficiency.

**Figure 1:**
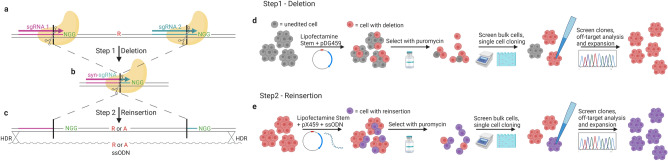
CRISPR Del/Rei overview and workflow. (**a**) In Step 1, two sgRNAs (pink and blue) mediate a deletion containing the target base (red, “R” for reference allele) and portions of the protospacers (pink and blue). Importantly, one PAM site (green) is left external to the deletion. (**b**) Once the deletion is made, the protospacers are destroyed which prevents further Cas9 recruitment and cleavage, resulting in high editing efficiency. In Step 2, the sequence spanning the deletion junction (black vertical line) is used as a protospacer for a synthetic sgRNA (syn-sgRNA, pink and blue). The syn-sgRNA coupled with the spared PAM site mediates reinsertion of the deleted sequence, including any desired allele of the target base (“A” for alternate allele). (**c**) Once the sequence is reinserted by HDR, the syn-sgRNA protospacer is destroyed, again preventing additional Cas9 cleavage and yielding high editing efficiency. (**d**) Deletion workflow with transfection of pDG459. (**e**) Reinsertion workflow with transfection of pX459 and ssODN.

The CRISPR Del/Rei workflow (Fig. 1d–e) is simple and optimized for iPSCs. sgRNAs are cloned in one step^14^ into the all-in-one vectors pDG459 (Step1) or pX459 (Step 2). iPSCs are plated across multiple wells and transfected in replicates with Lipofectamine Stem. Transfected cells undergo positive puromycin selection, which is cheap and reduces the risk of cellular stress, death and contamination compared to cell sorting. After selection, editing efficiency is determined in the “bulk”- cell populations from each transfected well- by simple PCR amplification and electrophoresis. Wells with the highest efficiency are sparse plated for single-cell cloning and again screened for deletions (Step 1) or insertions (Step 2) (Fig. 2, Supplementary Fig. 1–5) by PCR-electrophoresis and confirmed by Sanger sequencing. Selected clones from Step 1 are then screened for off-target edits also by Sanger sequencing before advancing to Step 2. In Step 2 different alleles are reinserted in separate transfections or simultaneously through equimolar ssODNs mixes (Supplementary Fig. 1–4) and after the desired number of single cell clones are identified they are screened for off target editing. The whole process takes ~ 2 months.

**Figure 2:**
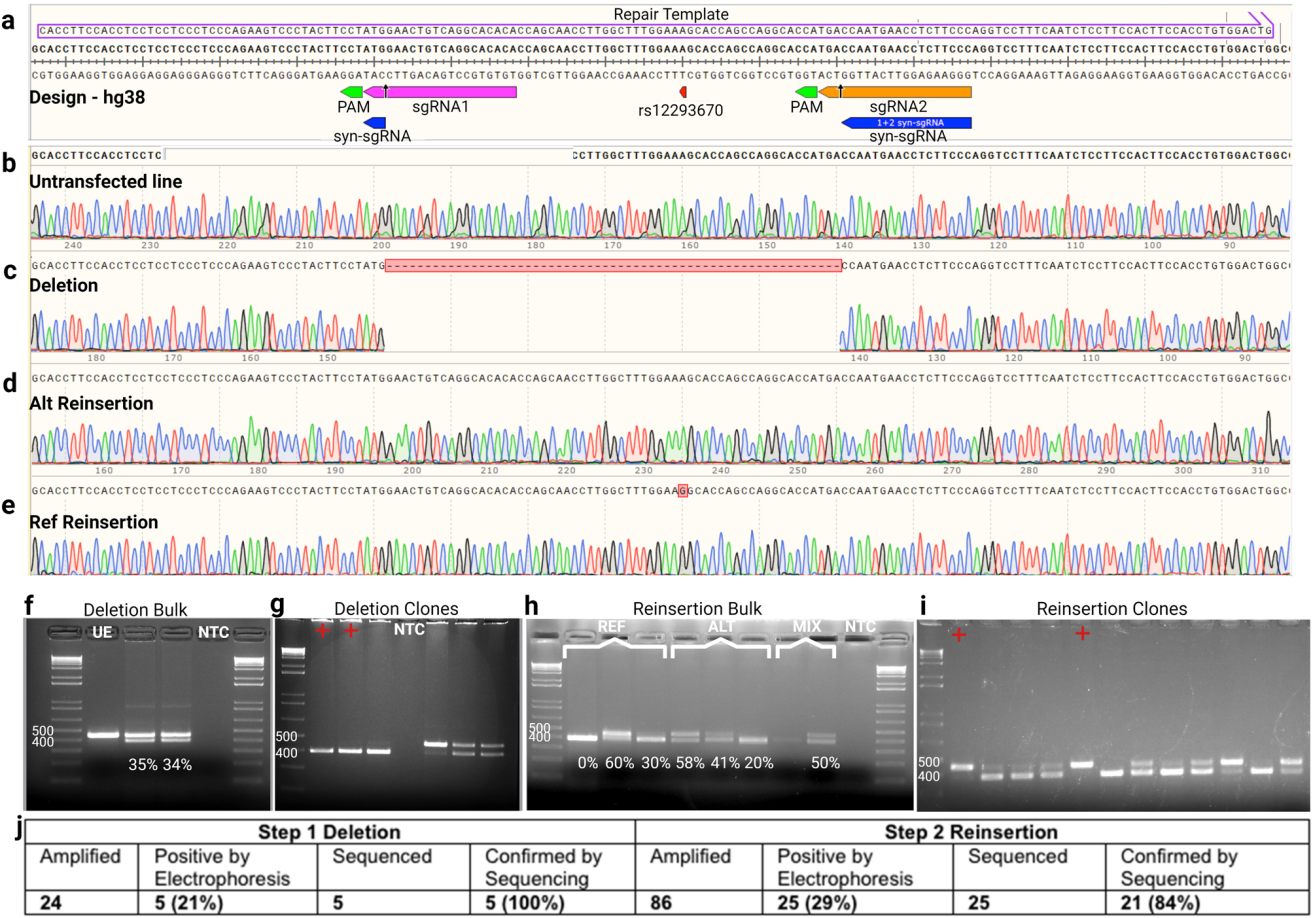
CRISPR Del/Rei of rs12293670 (*NRGN*) in the RU02 iPSC line. (**a**) sgRNA, syn-sgRNA, and ssODN repair template design. (**b**-**e**) Sanger sequencing chromatograms of the cell line before and after each step of editing. (**f**) Screen of the bulk cells from each deletion transfection well. Each lane represents a technical replicate from a separate transfection well unless otherwise marked. Editing efficiency quantified by Image J shown under each lane. (**g**) Example deletion clone screening gel. Each lane represents one clone. Lanes marked with a red cross were positive clones confirmed by Sanger sequencing. (**h**) Screen of the bulk cells from the reinsertion transfection. (**i**) Example screen of reinsertion clones derived from single-cell cloning. (**j**) Summary of total clones screened for each step. The positive by electrophoresis percentage refers to the number of clones that looked positive by gel out of the total clones amplified. The confirmed by sequencing percentage refers to the number of clones that were confirmed positive by Sanger sequencing out of the total number of clones sent for sequencing. Photographs of original gels available in Supplemental Fig. 6. All ladders are 1 Kb plus. UE = unedited/reinsertion positive control, NTC = no template negative control.

Using Step 1 we created three distinct homozygous and one heterozygous deletion (45–67 bp) across five different iPSC lines of three schizophrenia-associated intronic SNPs: rs12293670 in *NRGN*; rs4129585 in *TSNARE1*, and rs4766428 in *ATP2A2* (Fig. 2, Supplementary Figs.1–6, Supplementary Tables 1, 2, and 5). The average deletion efficiency across all loci was 35%, but varied significantly (0–100%) not only between loci but also between technical replicates which we cannot explain. We routinely performed 3 or more technical replicates in different wells and chose the best one to proceed, which we recommend for both steps. Single cell clones that were positive by electrophoresis (43% on average) were confirmed by Sanger sequencing (81% on average). Among candidate off-target sites, selected to have 3 or more mismatches with the target sequence and lay in transcribed DNA or open chromatin, we found no inadvertent editing out of 205 instances across four experiments (Supplementary Table 3). Using Step 2 we performed reinsertions at the three homozygously deleted loci across four different iPSC lines (Supplementary Tables 1, 2, and 5, Supplementary Figs. 1–4). For each experiment, one off-target-free deletion clone was chosen. Syn-sgRNAs were designed along with 160 bp ssODN repair templates for HDR with homology arms flanking the deletion; one ssODN for each variant allele (Supplementary Table 1). The two ssODNs were either introduced in separate transfections or simultaneously in an equimolar mix (Supplementary Figs. 1–4). The bulk reinsertion efficiency average was 25% again with significant variation between loci and intra-locus technical replicates (0%–63%; Fig. 2, Supplementary Figs. 1–4). After single-cell cloning on average 25% of clones showed re-insertion by electrophoresis (Fig. 2, Supplementary Figs. 1–4) and sequencing confirmed 60% of those. Mixed ssODNs generated both heterozygous and homozygous clones (Supplementary Fig. 2). One round of sib-selection ^15^ increased the bulk efficiency and percentage of positive clones up to 100% (Supplementary Fig. 3 and 4), which greatly reduced but did not eliminate the need for sparse plating and colony picking needed to acquire clean clones. Reinsertion clones were screened for off-target editing as in Step 1. Off-target editing was detected at only 5/449 possible instances (1.1%) and was restricted to two candidate sites of the *TSNARE1* syn-sgRNA (Supplementary Table 3).

Advantages of CRISPR Del/Rei include high efficiency in iPSCs and identical experimental procedures for the generation of different alleles in isogenic clones. It is flexible in terms of the size and location of the deletion, allowing the targeting of loci that are repetitive or lack PAM sites near the target. When non-coding variants are tested, the deletion can provide a useful first screen for functionality. Heterozygote edits can also be generated either by generating a heterozygote deletion in Step 1 (Supplementary Figure 5) or by introducing a mixed HDR template in Step 2. Finally, more complex modifications can be introduced since the re-inserted sequence can carry as many modifications as one might want.

The transfection and selection protocols are flexible and can be modified to each laboratory’s preferred methods. Strategies to further increase HDR efficiency may be incorporated if desired^9, 16^. CRISPR Del/Rei was designed and applied in iPSCs, which are difficult to transfect and edit. In more commonly used cancer-derived or immortalized cell lines higher efficiencies are expected. As with most editing approaches, efficiency varies between transfections, across cell lines, and for different target sites. There is likely increased risk of off-target editing due to the use of 3 sgRNAs, however, our data suggests this is not a significant problem (Supplementary Table 3). Additionally, only one deletion clone is required to complete Step 1, therefore off-target effects can be mitigated by selecting an off-target-free clone. Introducing large deletions (> 110 bp) is not ideal because the reinsertion would require the use of plasmids as HDR templates, which are less efficient than ssODNs^17^. Our design requires having 2 PAM sites within 110 bp, including one that is external to the cut site, which may be a challenge in some genomic regions but was never a limitation in our experiments. It must be noted that CRISPR Del/Rei is not an appropriate strategy for therapeutic in vivo editing because of the two steps and clone isolation involved. It could however be used for editing cells in vitro and re-introducing them to the patient if appropriate.

CRISPR Del/Rei is a novel, effective strategy for quick and efficient scarless editing. We find it especially advantageous for generating isogenic cell lines to study disease-associated variants. We believe that in combination with cell differentiation to specific cell types or the generation of organoids, it can provide a significant benefit to future research.

## Supplementary Information

Supplementary Information.
